# Neuroimaging Studies of the Neural Correlates of Heart Rate Variability: A Systematic Review

**DOI:** 10.3390/jcm12031016

**Published:** 2023-01-28

**Authors:** Patrycja S. Matusik, Chuwen Zhong, Paweł T. Matusik, Omar Alomar, Phyllis K. Stein

**Affiliations:** 1 Department of Diagnostic Imaging, University Hospital, 30-688 Kraków, Poland; 2 Center for Social Epidemiology and Population Health, Department of Epidemiology, University of Michigan School of Public Health, Ann Arbor, MI 48109, USA; 3 Department of Electrocardiology, Institute of Cardiology, Faculty of Medicine, Jagiellonian University Medical College, 31-202 Kraków, Poland; 4 Department of Electrocardiology, The John Paul II Hospital, 31-202 Kraków, Poland; 5 Department of Internal Medicine, Cardiovascular Division, Washington University School of Medicine, Saint Louis, MO 63110, USA

**Keywords:** heart rate variability, heart–brain connections, brain structures, brain activity, resting state, magnetic resonance imaging, neuroimaging

## Abstract

Direct and indirect links between brain regions and cardiac function have been reported. We performed a systematic literature review to summarize current knowledge regarding the associations of heart rate variability (HRV) and brain region morphology, activity and connectivity involved in autonomic control at rest in healthy subjects. Both positive and negative correlations of cortical thickness and gray matter volumes of brain structures with HRV were observed. The strongest were found for a cluster located within the cingulate cortex. A decline in HRV, as well as cortical thickness with increasing age, especially in the orbitofrontal cortex were noted. When associations of region-specific brain activity with HRV were examined, HRV correlated most strongly with activity in the insula, cingulate cortex, frontal and prefrontal cortices, hippocampus, thalamus, striatum and amygdala. Furthermore, significant correlations, largely positive, between HRV and brain region connectivity (in the amygdala, cingulate cortex and prefrontal cortex) were observed. Notably, right-sided neural structures may be preferentially involved in heart rate and HRV control. However, the evidence for left hemispheric control of cardiac vagal function has also been reported. Our findings provide support for the premise that the brain and the heart are interconnected by both structural and functional networks and indicate complex multi-level interactions. Further studies of brain–heart associations promise to yield insights into their relationship to health and disease.

## 1. Introduction

**Figure 1:**
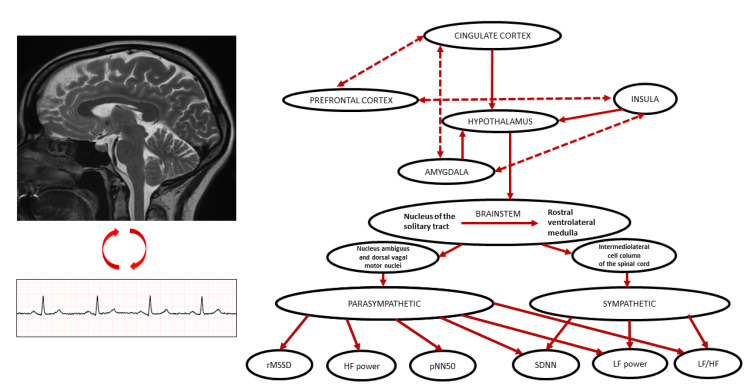
A simplified representation of the connections between neural structures and heart rate variability measures. Based on: [3,4]. Abbreviations: HF power—high frequency power; LF power—low frequency power; pNN50—percentage of successive NN intervals that differ by more than 50 ms; rMSSD—root mean square of successive variances of NN intervals; SDNN—standard deviation of NN intervals.

The connection between the functioning of the brain and the heart was first described in 1865 by Claude Bernard, the French physiologist [1]. His finding that the vagus nerve connects the brain and the heart is considered to be the basis for contemporary neuroscience. However, the structures that are actually involved in this linkage and the determinants of their effect are only now being elucidated [2]. Figure 1 depicts a simplified representation of the connections between neural structures and heart rate variability (HRV) measures.

Currently, two main theories exist regarding cardiac vagal control: polyvagal theory and the neurovisceral integration model [1,5]. Polyvagal theory specifies two functionally distinct branches of the vagus nerve, the evolutionarily newer ventral vagal complex (found in mammals) and the evolutionarily older dorsal vagal complex (found in both reptiles and mammals) [5,6]. The ventral vagal complex has lower motor neurons in the nucleus ambiguus and the dorsal vagal complex originates in the dorsal motor nucleus. The ventral vagal complex dominates in healthy people, inhibiting the dorsal vagal complex and sympathetic nervous system activity, contributing to feeling safe, sleeping and engaging in social interactions. Thus, the polyvagal theory provides us with understanding of the neural circuits in lower structures of the brainstem that support different kinds of behaviors.

Although the neurovisceral integration model also includes brain structures above the brainstem and indicates that emotional and cognitive functions are controlled by brain areas which are also associated with the control of autonomic outflow, this model states that the functioning of prefrontal-subcortical inhibitory circuits, important for self-regulation, is linked with the heart via the vagus nerve and that higher vagal tone results in better cognitive function, emotional balance and health [7]. The neurovisceral integration model proposes that the prefrontal amygdala pathways are related to HRV and contribute to individual differences in resting HRV. In particular, inhibitory processes from the prefrontal cortex to the amygdala are crucial for HRV modulation [8].

A variety of human imaging studies have investigated the relationship between different anatomical structures and electrocardiographic parameters, including HRV [9]. HRV, which is calculated from the intervals between normal heart beats, is a non-invasive method for assessment of ANS function [3,10,11]. Abnormalities of autonomic input to the heart are generally reflected in decreased HRV [12,13,14,15,16]. However, the assumption that increased HRV is always a marker for better ANS functioning ignores the fact that the organization of the inter-beat intervals is also a component of health [17]. For example, someone in atrial fibrillation has extremely high HRV, but the completely disorganized rhythm is not a marker for health [18]. Increased heart rate and impaired circadian HRV are related to higher mortality in patients with atrial fibrillation [19,20,21]. Similarly, higher resting heart rate in patients with heart failure and sinus rhythm is generally associated with increased mortality [22,23]. Both branches of ANS, the parasympathetic (PNS) and the sympathetic (SNS) modulate HRV and generally exert opposing effects on the heart rate (Figure 1) [24]. However, changes in PNS input to the sino-atrial (SA) node have effects on heart rate that are immediate (the next beats) and occur more rapidly than changes in heart rate due to changes in SNS input to the SA node, because PNS binding to the pacemaker cells of the SA node is mediated by direct coupling of vagal fibers via acetylcholine, whereas binding of signals from the SNS via norepinephrine occur by a slower, second messenger system [25]. Importantly, intrinsic cardiac ganglionated plexi are involved in vagal control of the SA node (mainly between the aortic root and the medial wall of the superior vena cava and between the anterior antrum of the right superior pulmonary vein and the superior vena cava) and atrioventricular node (mainly between the posterior wall of the coronary sinus ostium and the left atrium and in the superolateral area around the root of the left superior pulmonary vein) and might be potential target for cardio-neuroablation [26]. Furthermore, the supine resting state has very little SNS outflow, so vagally mediated HRV (e.g., respiratory sinus arrhythmia), is more likely to correlate with neuroimaging results [27] and longer-term HRV, which is generally associated with outcomes, cannot be measured while a patient remains in the scanner [28]. Definitions for HRV measures used in the neuroimaging studies cited in this review are listed in Table 1.

The morphology of the grey matter and its relationship to HRV can be evaluated using T1-weighted magnetic resonance imaging (MRI) with volumetric comparisons of manually, semi-automatically or automatically delineated neuroanatomical regions of interest, or with automated computerized techniques such as voxel-based morphometry (VBM) and cortical thickness measures [34]. The VBM method hypothesizes that the gray matter volume of each brain region can be quantified by calculating the sum of all the voxels classified as gray matter [35]. Assessment of cortical thickness with T1 -weighted images is a methodological alternative to VBM measurements, especially in detection of small cortical changes [34].

The relationship between *brain activity* in different regions and HRV can be evaluated using functional magnetic resonance imaging (fMRI). This method shows time-varying regional changes in brain metabolism, which can arise from different task-induced cognitive and emotional state changes or be the result of changes in the brain during the resting state [36]. Changes in blood flow and blood oxygenation in the brain are closely linked to neural activity and are called “hemodynamic responses.” Neuronal activity causes an increase in local cerebral blood flow (CBF) and oxygenated blood displaces deoxygenated blood and generates the blood oxygen level dependent (BOLD) MRI signal. Importantly, both vascular and neural mechanisms underlie the BOLD signal [37]. Therefore, in addition to reflecting neural activity, the BOLD signal also reflects changes in other hemodynamic responses such as CBF, cerebral blood volume and the cerebral metabolic rate.

The key component of the BOLD signal is a hemodynamic response function (HRF), measurable as the transient signal change arising after a short stimulus and represents a fixed coupling between neural activity and the subsequent local hemodynamic response. The temporal structure of the HRF differs across brain regions and between subjects [38]. These interindividual differences can be investigated by three parameters that characterize the HRF: response height (RH), time-to-peak (TTP) and full-width at half-max (FWHM). Alterations in CBF can be evaluated using an injected contrast agent and perfusion weighted MRI or using arterial spin labeling, which allows real time, non-invasive, quantitative assessment of cerebral perfusion [39]. The main postulate of CBF assessment, using arterial spin labeling, is that what is measured is the delivery of blood to the capillary bed and, hence, a property of the tissue itself. This method is characterized by a poorer signal-to-noise ratio (a measure that compares the level of a desired signal to the level of background noise) than BOLD-based fMRI. However, in some cases, this method is favored over BOLD-based fMRI, for example, for tasks, which are sporadic or longer than a few minutes.

Investigation of neural correlates of resting HRV are important, because HRV at rest is a commonly used surrogate for the functioning and health of the autonomic nervous system and the resting state is the least confounded. Thus, the aim of this review was to summarize current knowledge of the associations of HRV and brain regions involved in autonomic control during the resting state in healthy subjects.

## 2. Materials and Methods

**Figure 2:**
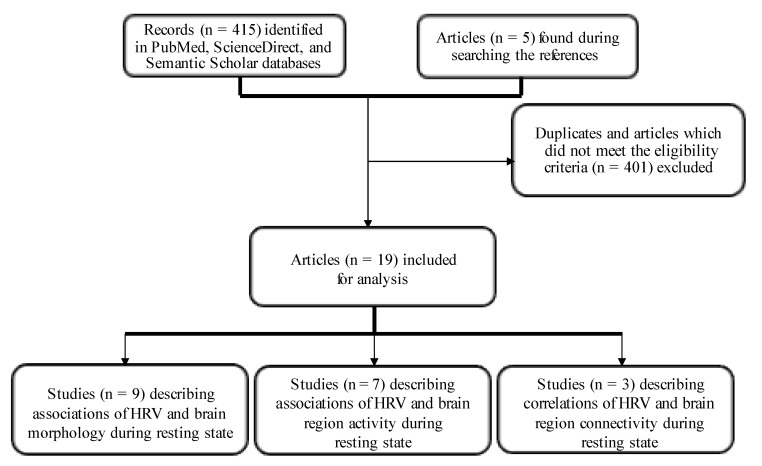
Flow chart of systematic review of studies on neural correlates of heart rate variability during the resting state. Abbreviations: HRV—heart rate variability.

A systematic literature review was conducted consistent with Preferred Reporting Items for Systematic Reviews and Meta–Analyses (PRISMA) guidelines [40]. Detailed methodology is depicted in the flow diagram in Figure 2.

### 2.1. Information Sources and Search Strategy

PubMed, Semantic Scholar and ScienceDirect databases were explored using the following keywords: (“heart rate variability” OR HRV) AND (MRI OR “magnetic resonance”) AND (brain OR “brain function” OR “brain structure”) AND (rest OR “resting state”).” Records found using these keywords were then evaluated to determine if they fit criteria for inclusion in the current review. The initial search identified 415 results. After removing duplicates, the initial eligibility assessment was based on titles and abstracts. Then, the full texts that fit the inclusion criteria were screened for the eligibility criteria. Two authors (P.S.M. and C.Z.) independently examined the full texts to confirm the suitability of the studies for the following qualitative synthesis. During the whole process, disagreements were resolved by consulting other co-authors (P.T.M. and O.A.) and supervisor (P.K.S.). The last search was conducted on 15 August 2022.

### 2.2. Study Selection and Eligibility Criteria

Studies describing the association of HRV with brain regions involved in ANS control during the resting state in healthy subjects were selected. Only studies assessing neural correlates with HRV using structural or fMRI were included. Studies describing subjects with diseases affecting the brain, or that explored changes in association with different tasks were excluded. Studies that included healthy controls for comparison were excluded when they did not report the measures we reviewed here. Studies performed on newborns or children were also excluded. Only articles written in English and accessible in full-text format were included. Three studies in which HRV measurements were not made during MRI scan or immediately before/after were excluded. Five additional articles were found while searching references. In the end, N = 420 records were found and 19 articles reporting on HRV and brain imaging during the resting states in healthy adults were selected and analyzed for this review.

### 2.3. Reporting of Findings

The following data were extracted from each analyzed study: authors, year of publication, characteristics of participants (including age, gender), methodology and type of HRV measure, methodology of the imaging modality and results of the study. Findings are summarized in Table 2, Table 3 and Table 4.

## 3. Results

### 3.1. Associations of HRV with Brain Morphology or Structural Covariates at Rest (Table 2)

There were N = 9 studies examining the associations of HRV and brain morphology or structural covariates during the resting state in healthy individuals [2,41,42,43,44,45,46,47]. Mean inter-beat interval (IBI) time series, high frequency (HF) power, low frequency (LF) power and the square root of the mean of the squares of variances between adjoining NN intervals (rMSSD) were investigated. The majority of the studies of associations between brain structure and HRV reported that brain regional anatomical measurements, such as cortical thickness or volume, correlated positively with HF power and rMSSD. Furthermore, most of the correlations between brain structures and HRV were found for right-sided brain structures. Details of these studies are described below.

Winkelmann et al. [41], in a study involving 30 young, healthy subjects, observed that HF power (measured in a sitting position during a 10-min resting phase) correlated significantly with the thickness of an area located primarily within the cingulate cortex (CC) in both the right (rH) and left (lH) hemispheres. This area is termed the caudal anterior cingulate cortex (ACC) in the Desikan atlas (a brain atlas based on an automated system for subdividing the human cerebral cortex into standard gyral-based neuroanatomical regions [48]). In addition, positive correlations between mean cortical thickness and HF power were observed for the inferior temporal gyrus in the lH, the supramarginal gyrus in the lH, the lingual gyrus in the rH, the pars triangularis in the rH, the precentral gyrus in the rH, the rostral middle frontal gyrus in the rH, the superior frontal gyrus in the rH, the superior temporal gyrus in the rH and the transverse temporal cortex in the rH. The only negative correlation was observed between HRV and the isthmus CC in the lH. Results of this study suggested that right-sided brain structures might be especially important for regulation of resting HRV.

Wei et al. [44] in a study of 185 healthy participants delineated a structural covariance network of the amygdala using MRI-based gray matter volume (GMV). The structural covariance network describes the existence of positively correlated brain regional anatomical measurements, such as cortical thickness or volume, between pairs of brain regions and permits the examination of covariation of gray matter morphology between brain regions and across individuals. The IBI time series was generated from a photoplethysmography signal and rMSSD was calculated. They observed that structural correlations from the right amygdala to the dorsal medial prefrontal cortex (dmPFC)/dorsal ACC expanding into medial motor regions (i.e., pre-supplementary motor area (SMA)/SMA) were positively correlated with rMSSD. Moreover, participants with higher values for rMSSD had stronger structural correlations between the right amygdala and dmPFC/dorsal ACC, when compared to participants with decreased rMSSD. Consistent with results reported by Winkelman et al. [41] Wei’s group did not find any significant correlation between the left-sided amygdala-specific covariance network and rMSSD.

Wei et al. [43] also used the covariance analysis of MRI-based GMV to investigate the relationship between structural covariance of the salience network [SN, which plays a role in selecting which stimuli are deserving of our attention [49]] and an individual’s vagally-mediated HRV levels. In this study two independent cohorts of participants (N = 222) were used: *The Leipzig Study for Mind-Body-Emotion Interactions* (LEMON) and the *Enhanced Nathan Kline Institute-Rockland Sample (NKI-RS).* The IBI time series were derived from photo-plethysmographic signals and rMSSD was calculated. Positive structural correlations with rMSSD and the left anterior insula to the bilateral orbitofrontal cortex (OFC), dorsal ACC, left medial prefrontal cortex (mPFC), right inferior lobe and Precuneus (PCu) were noted in the LEMON sample. In the NKI sample, positive structural correlations with rMSSD and the left anterior insula to the right OFC, mPFC, anterior insula and left dorsal ACC were found. The structural correlation from the left anterior insula to the dorsal ACC was also positively related to rMSSD on the conjunction maps in these two sample groups. The pooled data analysis demonstrated that structural correlations from the left anterior insula to the bilateral dorsal ACC and PCu and from the right anterior insula to right OFC were positively related to rMSSD.

In a third study, the same investigators [42] examined the association of HRV and GMV in brain regions of the central autonomic network (CAN, i.e., the striatum, amygdala, insula, superior temporal gyrus and para-hippocampal gyrus). The IBI time series were, again, generated from a photoplethysmography signal and HF power, LF power and rMSSD were calculated. The investigators showed that HF power was negatively correlated with GMV in the right putamen, amygdala, insula, caudate, temporal pole, superior temporal gyrus and para-hippocampal gyrus. No significant positive correlation was found between GMV and HF power in these studies. Similar results were found for rMSSD and LF power. Gender and age effects on the associations of HF power with GMV were also studied. Before correction for cluster level false discovery rate, only the putamen showed gender difference at an uncorrected level (*p* < 0.05), indicating that the striatal function may reflect gender effects on parasympathetic modulation of the heart. However, no significant relationship with gender or age was seen after cluster-level false discovery rate correction. The lack of association with age may have been a result of the fact that adults >60 years were not included in this study.

Yoo et al. [2] investigated the effect of age on the relationship between resting HRV and cortical thickness in a group consisting of 19 younger adults and 19 older adults. A baseline measure of HRV was provided during a pre-scan period lasting 3 min. IBIs were derived from the ECG signal and rMSSD was calculated. A positive correlation was observed between rMSSD and cortical thickness of the lateral OFC in both the rH and lH. However, this correlation was significant for the rH only in the older subgroup of participants. Moreover, for the same subgroup of older individuals, a significant correlation of rMSSD with thickness of the pars orbitalis in the rH was also found, while only a trend towards significance was observed between the rostral ACC in the rH and rMSSD in the younger subgroup. However, this sample was relatively small (N = 38) and a larger study is needed to definitively explore these relationships.

Kumral et al. [46] also investigated the effect of age on the associations between HRV and GMV in a study performed on 388 healthy subjects. rMSSD was acquired from the ECG recordings (10 s in the LIFE—*Leipzig Research Centre for Civilization Diseases* and 4 min in LEMON—the *Leipzig Study for Mind-Body-Emotion Interactions* study). They observed no significant association between rMSSD and GMV when all subjects were taken together. However, in the middle-aged group there was a significant rMSSD-related increase of GMV in the left cerebellum.

Wood et al. [33], in a study of 55 healthy participants, demonstrated that cortical atrophy in the frontal lobe is related to both the sympathetic and parasympathetic changes occurring with age. R–R intervals were acquired during 10 min in supine position and HF power, the standard deviation of the width of the Poincaré plot (SD1) and total spectral power were calculated. They observed that cortical thickness correlated with SDNN and total spectral power over the right hemisphere and bilateral MPFC. SD1 was correlated with cortical thickness at the left MPFC. Generally, the relationship between cortical thickness and HRV was influenced by age. However, independent of age, the cortical thickness of the left MPFC was a dominant predictor of HRV derived from a standard three-lead ECG.

Finally, Koenig et al. [47], performed a cross-sectional pooled mega analysis of 1218 participants and demonstrated that rMSSD (calculated from either ECG or plethysmography recordings) was associated with cortical thickness, when accounting for age, for the left and right lateral OFC. After adjusting for all potential confounds (including research group, BMI, age, sex, and sexXage), they observed significant associations between the cortical thickness of the lateral OFC bilateral, right medial OFC, right insula and left insula and rMSSD. Exploratory analysis of all 34 regions of interest (ROIs) in both hemispheres showed significant associations between rMSSD and cortical thickness in several regions. However, only the association between rMSSD and cortical thickness in the left lateral OFC remained significant after false discovery rate correction of *p*-values.

In contrast with the studies described above, Fridman et al. [45], did not observe any associations between cortical thickness in selected CAN regions and HF power during the resting state. In this study, involving 127 young women, associations between cortical thickness in the anterior midcingulate cortex (MCC), pregenual and sub-genual ACC, OFC and anterior insula and HF power calculated from the ECG were investigated. Only during stress tasks, as opposed to during the resting state, were the right pregenual and sub-genual ACC thicknesses positively associated with peak change in HF power during the 10-min stressor period.

**Table 2:** Findings of published cross-sectional studies describing associations of heart rate variability and brain morphology or structural covariates during the resting state.

Study	Group	HRV Parameters and Methodology	MRI Methodology	Main Brain Regions Associated with HRV	Positive (+) or Negative (-) Correlations of HRV with Different Brain Regions	Studied (+) or Not (-) Age or Gender-Dependent Associations between HRV and Different Brain Regions
		HF	1.5T MRI			
Winkelmann et al., 2016 [42]	N = 30 young participants (8 female, mean age: 22.5 ± 3.9 years)	(3- leads ECG recorded in a sitting position during a 10-min resting phase, then five artifact-free minutes analyzed)	(thickness of cortical surfaces and volume of subcortical brain structures)	Positive correlations with mean cortical thickness of caudal ACC (R), LG (R), pars triangularis (R), precentral gyrus (R), rostral MFG (R), SFG (R), superior TG (R), transverse temporal cortex (R), caudal ACC (L), inferior TG (L) and SMG (L). Negative correlation with isthmus CC (L).	+/-	-
		rMSSD	3T MRI			
Wei et al., 2018 [44]	N = 185 from NKI-RS (95 female, mean age: 35.2 ± 14.0 years)	(IBI time series derived from PPG)	(GMV, the voxel-based morphometry analysis)	Structural correlations from amygdala (R) with dorsal mPFC (L)/dorsal ACC (R) extending into pre-SMA/SMA (R).	+	-
		rMSSD	3T MRI			
Wei et al., 2021 [43]	N = 114 from NKI-RS (47 female, age 36.1 ± 13.3 years) N = 108 from LEMON (31 female, mean age 41.4 ± 20.7 years)	(IBI time series derived from PPG)	(GMV, the voxel-based morphometry analysis)	Positive structural correlations from anterior insula (L) to bilateral OFC, dorsal ACC, mPFC (L), inferior lobe (R) and Precuneus (in the LEMON sample). Positive structural correlations from anterior insula (L) to OFC (R), mPFC (R), anterior insula (R) and dorsal ACC (L) (in the NKI sample). Positive structural correlation from anterior insula (L) to dorsal ACC on the conjunction maps. Positive structural correlations from anterior insula (L) to bilateral dorsal ACC and PCu and from anterior insula (R) to OFC (R) in the pooled data analysis.	+	-
		HF, rMSSD, LF	3T MRI			
Wei et al., 2018 [44]	N = 185 from NKI-RS (95 female, mean age: 35.2 ± 14.0 years)	(IBI time series derived from PPG)	(GMV, the voxel-based morphometry analysis)	HF: Negative correlations with GMV in putamen (R), caudate (R), amygdala (R), insula (R), superior temporal gyrus (R), temporal pole (R), para-hippocampal gyrus (R). rMSSD: Similar results obtained for HF power LF power No significant correlations	-	+
		Mean IBI, rMSSD	3T fMRI			
Yoo et al., 2018 [2] *	N = 19 older adults (9 female, age range: 62–78 years) N = 19 younger (7 female, age range:19–37 years) *	(IBIs derived from the ECG signal during a 3-min pre-scan	(cortical reconstruction and volumetric segmentation)	Mean IBI: Negative correlations with caudal ACC (L) in all subjects. rMSSD: Positive correlations with lateral OFC (L) (R) in the entire group and with lateral OFC (R) and pars orbitalis (R) in older subjects. Rostral ACC (R) trended to significance in the younger group.	+/-	+
		rMSSD	3T-fMRI			
Kumral et al., 2019 [46]	N = 388 (140 younger: 26.0 ± 4.2 years, 119 middle-aged: 46.3 ± 6.2 years, 129 older: 29 66.9 ± 4.7 years)	(ECG recordings, 10-s in LIFE and 4-min in LEMON study)	(GMV, the voxel-based morphometry analysis)	rMSSD: In the middle-aged group a significant rMSSD-related increase of GMV in the left cerebellum. No significant findings in the younger or older groups	+	+
		SDNN, SD1, HF, total power	3T-MRI			
Wood et al., 2017 [33]	N = 55 (21–73 years; 18 female)	(a standard three-lead ECG, 10-min recordings)	(a high-resolution T1-weighted structural volume was acquired with a 3D MPRAGE sequence)	SDNN, total spectral power: Cortical thickness correlated with SDNN and total spectral power over the right hemisphere, as well as the bilateral MPFC. SD1: correlated with cortical thickness at the left MPFC. HF power: correlated with the average cortical thickness in the right and left hemisphere, as well as the regions of interest, namely the bilateral MPFC and bilateral insula. Age influenced the relationship between cortical thickness and total power HF power and SD1. However, independent of age the thickness of the MPFC (L) was a dominant predictor of SDNN, total power and HF power (*p* = 0.05).	+	+
		rMSSD	3T-MRI			
Koenig et al., 2020 [47]	N = 1218 (50.5% female; mean age 36.7 [range: 12–87] years).	(both ECG and PPG recordings)	(cortical thickness of ROI in millimeters)	rMSSD: A decline in rMSSD, as well as cortical thickness with increasing age, especially in the OFC. After accounting for all potential confounds including: research group, age, sex and sex × age a significant relationship between cortical thickness of the lateral OFC (L,R), medial OFC (R), insula (R,L) and rMSSD. Exploratory analysis of all 34 ROIs in the right and left hemispheres revealed significant associations between rMSSD and cortical thickness in several regions. However, only the relationship between rMSSD and cortical thickness lateral OFC (L) remained significant after false discovery rate correction of p-value.	+	+
		HF	3T fMRI			
Fridman et al., 2020 [45]	N = 127 young women (mean age of 19.59 ± 0.49 years)	(30-min ECG recordings)	(cortical reconstruction and cortical thickness calculation)	None in the resting state.	None	-

Abbreviations: ACC—anterior cingulate cortex; AHAB-II—the Adult Health and Behavior Project, phase II; BOLD—blood-oxygen-level-dependent; CC—cingulate cortex; CG—cingulate gyrus; CORR—Consortium for Reliability and Reproducibility project; dlPFC—dorsolateral prefrontal cortex; dmPFC—dorsomedial prefrontal cortex; ECG—electrocardiogram; fMRI—functional magnetic resonance imaging; FOC—frontal orbital cortex; GCGC—globally conditioned Granger causality; GMV—gray matter volumes; HF—power in high frequency range; HRV—heart rate variability; IC—insular cortex; IFG—inferior frontal gyrus; LEMON—the “Leipzig Study for Mind-Body-Emotion Interactions”; LF—power in low frequency range; LG—lingual gyrus; LIFE—the “Leipzig Research Centre for Civilization Diseases”; L—left; LOC—lateral occipital cortex; MCC—midcingulate cortex; MFG—middle frontal gyrus; mPFC—medial prefrontal cortex; MRI—magnetic resonance imaging; NKI-RS—Nathan Kline Institute-Rockland Sample; nu—normalized units; PaCG—paracingulate gyrus; PIP—the Pittsburgh Imaging Project; POp—parietal operculum; PPG—photoplethysmography signal; R—right; rMSSD—square root of the mean of the squares of differences between adjacent NN intervals; ROIs—regions of interest; SD1—Poincaré plot standard deviation perpendicular the line of identity; SFG—superior frontal gyrus; SMA—supplementary motor area; SMG—supramarginal gyrus; SN—salience network; SOG—superior occipital gyrus; T—tesla; TR—repetition time. * For the study conducted by Yoo et al., 2018 data are shown only for subgroup 1. The ECG data obtained in subgroup 2 was averaged across different in-scanner task conditions (data from a resting state pre-scan not available).

### 3.2. Association of HRV and Brain Region Activity at Rest (Table 3)

We found N = 7 studies investigating the association between HRV and brain region activity at rest in healthy participants [38,46,50,51,52,53,54,55,56]. Standard deviation of NN intervals (SDNN), rMSSD, HF and LF power were studied and the LF/HF ratio was calculated. ECG recordings were performed during scans. Interestingly, the majority of significant correlations of HRV and brain region activity were negative and were strongest for HF power. For LF power, however, mostly positive correlations were noted. Activity of brain structures from both right and left hemispheres correlated with HRV.

Valenza et al. [50], in a study of 34 young, healthy individuals observed a number of clusters whose functional brain activity (measured as BOLD signals) displayed negative correlations with both instantaneous LF and HF power fluctuations derived from R-R intervals extracted from a finger pulse signal. HRV was measured during four 15-min MRI scans. These areas involved, among others, cortical structures including the insular cortex and structures such as the thalamus, putamen, brainstem and right caudate (Table 3). No significant positive correlations were found between functional brain activity and either HF or LF power at rest.

In another study, a continuation of the previous one, Valenza et al. [51], studied brain areas functionally linked to HF power at rest. Consistent with results of the prior study, negative associations between HF power and the BOLD signal in different brain areas were found (Table 3). However, there was no significant association between HF power and resting-state activity in the amygdala or with other subcortical region such as the left pallidum, putamen and right caudate. This result seems inconsistent with a prior report [50], which was carried out on the same number of participants and did not differ significantly in terms of methodology. Additionally, in this study, as opposed to in the previous one, positive significant relationships of heart rate and BOLD signal were found in clusters including, among others, the left dmPFC, right ACC and right superior frontal gyrus.

Valenza et al. [52] also applied non-linear HRV analysis to the investigation of heart-brain connections at rest. A finger pulse-oximeter device was used to acquire R-R intervals and inhomogeneous point-process approximate entropy, sample entropy and instantaneous dominant Lyapunov exponents were derived. Non-linear HRV parameters capture the underlying organization or randomness of the inter-beat intervals, rather than the magnitude of the oscillations in HR. Interpretation of HRV parameters in non-linear domains is more difficult than either time or frequency domains and their physiological and clinical implications remain unclear [13]. For example, approximate entropy is a measure of the degree of irregularity or randomness within a series of data: smaller values mean greater regularity and greater values indicate more randomness and system complexity [57]. Results indicated negative correlations between these non-linear HRV parameters and the activity of certain brain regions (Table 3), suggesting a wider implication of different brain areas in the CAN at rest than previously reported. They demonstrated that the temporal, paracingulate and cingulate gyri were involved in regulating autonomic influence on heartbeat dynamics and correlated negatively with all of the above parameters. The majority of significant correlations were observed between the brain regions examined and the instantaneous dominant Lyapunov exponent. Interestingly, in this population, there were no significant correlations of these non-linear HRV parameters with brain regions, such as the insular cortex, putamen, thalamus, amygdala and right caudate, which are classical CAN regions related to ANS control in a task-specific manner.

Duggento et al. [53] collected 7T resting-state fMRI data in nine healthy volunteers to study the direct interactions between resting-state brain activity and the ANS. R-R intervals were recorded using a piezoelectric finger pulse sensor and HF power, LF power and the LF/HF ratio were calculated. Both bivariate Granger causality (GC) and the globally conditioned GC (GCGC) were used to study brain–heart interactions. In comparison with traditional bivariate GC causality, the GCGC determines whether the causal relationship from one brain region to another is direct or mediated by a third brain region and untangles high overflow between locally connected brain signals. The GCGC demonstrated significant direct brain-heart interaction between HF, LF and the LF/HF ratio and the activity of different brain regions (i.e., the right amygdala, left hippocampus and left posterior cingulate gyrus, respectively) (Table 3). When the bivariate GC approach was used, there were even more brain areas whose activity showed significant interaction with both HF, LF and the LF/HF ratio (i.e., the left middle temporal gyrus, left supplementary motor area and left paracentral lobule, respectively).

Pfurtscheller et al. [54] studied the link between spontaneous neural and vascular BOLD signals and HRV in a study of 25 young individuals. To address this question, they estimated phase-locking values between the precentral gyrus and the insula. Phase-locking values are an often-used approach to quantify synchronization between neural signals and allow discrimination between neural and vascular BOLD oscillations. An ECG was recorded inside the scanner, with standard channels used for the positioning of the electrodes. LF power was calculated. The correlation between neural BOLD and HRV was significant for the RH only during the first resting period and showed a trend for the LH during the movement period. There were no significant correlations between vascular BOLD and LF power. Results of this study suggest that right-sided brain structures may be especially important for regulation of HRV at rest. On the other hand, these findings may be related to MRI-related anxiety, which can affect BOLD oscillations and is greater at the beginning of the test.

In another study of 23 participants, the same investigators [55] discovered that decreases in MRI-related anxiety and increases of LF power between both resting states occurred in subjects. In this study, LF power was additionally assessed in two bands 0.06–0.1 Hz (LFa) and 0.1–0.14 Hz (LFb). Interestingly, they observed a difference in LFa and LFb power between individuals with greater and smaller decreases in anxiety from the first resting state to the second one. LFa power increased significantly in the greater anxiety change group, while both components (LFa power and LFb power) increased significantly in the smaller change group. Furthermore, a significant correlation with neural BOLD was observed only for the LFb band in the rH. This may suggest that central commands (beginning in the prefrontal cortex and modulating cardiac activity via the vagus nerve) are detected above 0.1 Hz and the component of LF power in 0.1–0.14 Hz band is related to a PNS influence.

Wu et al. [38] examined correlations of HRV and hemodynamic response function (components of the BOLD signal) in a study on 67 participants (17 female, mean age: 50.6 ± 20.8 years). To address this, the response height (non-normalized and normalized), time to peak and full width at half maximum were estimated. HRV analysis was performed on the IBI time series obtained during a resting state session scan and mean IBI, SDNN, rMSSD, LF power, HF power and LF/HF ratio were calculated. Importantly, this study revealed that results were dependent on the repetition times (TR), which is the amount of time between successive pulse sequences applied to the same slice. For TR = 0.645 s, only full width at half maximum was significantly and positively correlated with the mean IBI (in the midbrain, pons and surrounding areas; Table 3) and LF power (in the midbrain and cerebellum anterior lobe). Notably, these correlations remained significant without cardiac fluctuation correction in pre-processing procedures. For TR = 2.5 s, only the response height (both non-normalized and normalized) was significantly correlated with LF power and SDNN (Table 3). This study confirmed that short TR (i.e., <1 s) should be used to reduce aliasing (an effect that causes different signals to become indistinguishable) between cardiac and brain signals.

**Table 3:** Findings of published cross-sectional studies describing associations of heart rate variability and brain region activity at rest.

Study	Group	HRV Parameters and Methodology	MRI Methodology	Main Brain Regions Associated with HRV	Positive (+) or Negative (-) Correlations of HRV with Different Brain Regions	Studied (+) or Not (-) Age or Gender-Dependent Associations between HRV and Different Brain Regions
		Entropy analysis	3T fMRI (BOLD)			
Valenza et al., 2020 [52]	N = 34 young healthy individuals (within the framework of the Human Connectome Project)	Inhomogeneous point-process approximate (ipApEn), sample entropy (ipSampEn), instantaneous dominant Lyapunov exponents (IDLE). Finger pulse oximeter placed on a digit used for the estimation of HRV.	Resting state data acquired in N = 4 runs ~15 min each)	ipSampEn: Negative correlations between BOLD signals and instantaneous changes in the temporal gyrus, planum temporale, frontal orbital cortex, opercular cortex, paracingulate gyri and cingulate gyri. ipApEn: The same areas as for ipSampEn, with the addition of the temporal fusiform. IDLE: Negative correlations with paracingulate gyri, cingulate gyri, temporal gyrus, superior and middle frontal gyri, lateral occipital cortex, angular gyrus, precuneus cortex, frontal pole, intra-calcarine, supra-calcarine cortices, para-hippocampal gyrus and hippocampus (L).	-	-
		HF	3T fMRI (BOLD)			
Valenza et al.,2019 [51]	N = 34 young healthy individuals (within the framework of the Human Connectome Project)	(Finger pulse oximeter used for the estimation of HRV)	(Resting state data acquired in N = 4 runs ~15 min each)	HF: Negative correlations with dorsal middle insula (R), paracentral lobule (R), Pop (R), posterior insula (L), bilateral anterior insula, bilateral medial dorsal and ventrolateral posterior thalamic nuclei, anterior MCC and posterior MCC/medial frontal gyrus/pre-SMA, primary motor cortex, superior TG, primary visual cortex, fusiform gyrus, lateral occipital gyrus and cerebellar lobule VIIIA.	-	-
		LF, HF	3T fMRI (BOLD)			
Valenza et al., 2017 [50]	N = 34 young healthy individuals (within the framework of the Human Connectome Project)	(Finger pulse oximeter used for the estimation of HRV)	(Resting state data acquired in N = 4 runs of ~ 15 min each)	LF: Negative correlations with caudate (R), insular cortex, superior, middle and IFG, LOC, PaCG and CG, precuneus cortex, thalamus, putamen, pallidum, brainstem, hippocampus and amygdala. HF: Negative correlations observed with caudate (R), pallidum (L), brainstem, hippocampus (L), amygdala (L), insular cortex, superior, middle and IFG, LOC, precentral and TG, precuneus cortex, TFC, FOC, thalamus and putamen.	-	-
		HF, LF, LF/HF	7T fMRI			
Duggento et al., 2016 [53]	N = 9 healthy volunteers (age 28 ± 3 years)	(Cardiac pulsation recorded by a piezoelectric finger pulse sensor)	(BOLD)	HF: Significant were transverse temporal gyri (R), lateral part of middle frontal gyrus (R), superior temporal pole (R), superior parietal lobule (R), amygdala (R), middle temporal gyrus (L), superior caudate nucleus (L), middle cingulate (L), brainstem, lobule III, IV, V of vermis, lobule IV, V of cerebellar hemisphere (R and L). LF: Significant were lobule IX of cerebellar hemisphere (R), posterior cingulate gyrus (L) and medial part of the superior frontal gyrus (L). LF/HF: Significant were lobule IV, V of cerebellar hemisphere (R), lobule X of vermis (nodulus), dorsolateral superior frontal gyrus (R), para=hippocampal gyrus (R), paracentral lobule (L), precuneus (L), hippocampus (L) and dorsolateral superior frontal gyrus (L), brainstem, lobule IV, V of vermis, lobule VI of cerebellar hemisphere (R and L), medial part of superior frontal gyrus (R and L).	Not studied (GCGC used to studying brain–heart networks)	-
		LFa (0.06–0.1 Hz), LFb (0.1–0.14 Hz)	3T MRI (BOLD)			
Pfurtscheller et al., 2018 [55]	N = 23 From 25 individuals (12 female, mean age 24 ± 3.2 years) two were excluded due to cardiac arrhythmia	(standard channels used for the positioning of the ECG electrodes) in two bands:	(Rest period followed by two movement sessions and a second rest period)	LFb: A significant correlation of neural BOLD between the precentral gyrus and the insula was discovered only for the LFb band in the right hemisphere (R).	+	-
		LF	3T MRI (BOLD)			
Pfurtscheller *et al.,* 2017 [54]	N = 25 individuals (12 female, mean age 24 ± 3.2 years)	(Standard channels used for the positioning of the ECG electrodes)	(rest period followed by two movement sessions and a second rest period)	LF: Correlation between neural BOLD and LF was significant for the right hemisphere (R) during both rest periods and showed a trend for the left hemisphere (L) during movement period. Participants with neural BOLD and longer phase-locking episodes between precentral gyrus and insula displayed greater HRV values.	+	-
		Mean IBI, SDNN, rMSSD, LF, HF and LF/HF ratio	3T fMRI (TR = 0.645s, TR = 2.5 s)	For TR = 0.645 s		
Wu et al., 2016 [38]	N = 67 from NKI-RS (17 female, mean age: 50.6 ± 20.8 years)	(IBI data from PPG)	A key component of the BOLD signal, the hemodynamic response function (response height, time to peak and full width at half maximum) were studied.	Mean IBI: in midbrain, pons and surrounding areas (culmen, para-hippocampal gyrus, thalamus, insula, superior temporal gyrus and dorsal anterior cingulate) correlated with the full width at half maximum LF: in midbrain and cerebellum anterior lobe correlated with the full width at half maximum For TR = 2.5 s LF: in MCC correlated with response height (non-normalized) and cuneus, precuneus, inferior parietal lobule, angular, precentral gyrus, ACC, medial/superior frontal gyrus and superior parietal lobule correlated with response height (normalized) SDNN: in MCC correlated with response height (non-normalized) and in cuneus, precuneus, inferior parietal lobule, angular, precentral gyrus, ACC, medial/superior frontal gyrus and superior parietal lobule, hippocampus, para-hippocampal gyrus, caudate, middle/inferior/superior temporal gyrus, supramarginal gyrus, postcentral gyrus and inferior/middle frontal correlated with response height (normalized).	+	-

Definitions for all abbreviations are shown in Table 2.

### 3.3. Associations of HRV and Brain Region Functional Connectivity at Rest (Table 4)

Three (n = 3) studies investigated associations between HRV and brain region functional connectivity during the resting state [32,46,58,59,60]. HRV was measured as rMSSD, HF power and LF power. ECG recordings were performed during scans. Significant positive correlations between HRV and brain region connectivity were observed. Connectivity of structures, especially from the right hemisphere were associated with HF power and rMSSD, both measures of vagal activity.

Chang et al. [32] in contrast with prior studies, investigated associations between HRV and not only the BOLD signal time series itself, but also brain functional connectivity in 35 young, healthy male subjects. Pulse oximetry was used for calculating rMSSD (across 45-s time windows), HF power and LF power. The right amygdala and dorsal ACC were chosen as the ROIs. They observed that increased rMSSD was positively related to increased functional connectivity between the dorsal ACC and regions including the CC, thalamus, basal ganglia, amygdala and midbrain and between the amygdala and regions including the CC, anterior insula, basal ganglia and dorsolateral prefrontal cortex (dlPFC). Fluctuations in HF power and LF power also correlated with the connectivity of both ROIs to the different brain regions (Table 4). Generally, stronger relationships of LF rather than HF power with functional connectivity for the dorsal ACC and amygdala for different brain regions were observed. However, it was shown that connectivity between the brainstem and posterior CC and the dorsal ACC had a significantly stronger correlation with HF power than with LF power. Regions in which only functional connectivity (not BOLD signal change) were associated with HRV included the dlPFC, the anterior insula and paracentral gyri. Additionally, they explored, between subjects, whether there were any brain areas whose mean functional connectivity were related to average measures of HF or LF power across a 10 min resting state scan. Brain regions were found that showed inter-subject differences across the entire scan of mean HRV and mean functional connectivity in ROIs including precuneus and subcortical structures, such as thalamus, putamen, pallidum and cerebellum.

Sakaki et al. [58] described the associations of HRV and functional connectivity between the right amygdala and medial prefrontal cortex (mPFC) across 17 younger and 18 older adults. The ECG was obtained from three leads and baseline measures of HRV were provided from 3 min pre-scan. They observed that greater rMSSD was related to increased connectivity between mPFC and right amygdala. A positive correlation between rMSSD and the mPFC-right amygdala connectivity was observed both in younger and older adults. Similarly, in the whole brain connectivity analysis, greater rMSSD was related to increased connectivity between the mPFC/ACC and right amygdala. Additionally, elevated HRV was related to increased connectivity between the right amygdala and ventrolateral PFC but only in younger adults. A similar, but weaker correlation was observed for the left amygdala. Other areas, in which connectivity with the amygdala were found to be correlated with rMSSD are listed in Table 4. Sakaki et al. also found positive correlations between both HF power, HF (nu), LF (nu) and the right amygdala-mPFC connectivity. Correlations with LF power were similar in direction but correlations did not reach statistical significance. This study suggests that resting vagally-mediated HRV may be useful in assessing the connectivity of the mPFC-amygdala regardless of age and thus, according to the neurovisceral integration model, may be a marker for the ability to regulate emotions.

Indirect functional connectivity of the dlPFC with an mPFC region was also demonstrated in a study on 271 participants performed by McIntosh et al. [60]. They showed that the connectivity between the left dlPFC and right MFG was associated with greater HF power collected using photoplethysmography. Functional connectivity between the right dlPFC and right superior frontal gyrus and bilateral middle frontal gyrus was associated with greater HF power. When sex differences were examined, the associations of HF power were similar with the functional connectivity between left dlPFC and right middle frontal gyrus and right dlPFC and right middle frontal gyrus in women only. Analyses performed on a subsample of 232 healthy individuals were consistent with whole-sample findings and demonstrated the associations of HF power and functional connectivity between the left dlPFC and right middle frontal gyrus and between right dlPFC and left middle frontal gyrus.

**Table 4:** Findings of published cross-sectional studies describing correlations of heart rate variability and brain regions connectivity during the resting state.

Study	Group	HRV Parameters and Methodology	MRI Methodology	Main Brain Regions Associated with HRV	Positive (+) or Negative (-) Correlations of HRV with Different Brain Regions	Studied (+) or Not (-) Age or Gender-Dependent Associations between HRV and Different Brain Regions
		rMSSD, HF and LF	3T fMRI			
Chang et al., 2013 [32]	N = 35 young, healthy male subjects	(the cardiac cycle monitored using a PPG placed on the right index finger)	(BOLD, scan duration of approx. 10 min)	HF: Connectivity between ROIs (amygdala (R), dACC) and thalamus and brainstem. LF: Connectivity between ROIs and parieto-occipital cortex. rMSSD: Connectivity between both ROIs and CC and basal ganglia (additionally, connectivity of dACC with the thalamus, amygdala (R) and midbrain and between amygdala (R) and the anterior insula and dlPFC)	+	-
		rMSSD, HF, LF, HF (nu), LF (nu)	3T fMRI			
Sakaki et al., 2016 [58]	N = 18 older adults (9 males, age range: 61–78 years) N = 17 younger adults (9 males, age range: 19–37 years)	(3-lead ECG activity recorded during the pre-scan of the mean duration lasted 3 min)	(BOLD, resting scan lasted 5.2 min)	rMSSD: connectivity between mPFC and amygdala (R), amygdala (R) and mPFC/ACC and amygdala (R) and vlPFC (similar, but weaker correlations for amygdala (L)) HF, HF (nu), LF (nu): Positive correlations with the mPFC-amygdala connectivity. LF: Trend towards significance in correlation with the mPFC-amygdala connectivity. Other regions, which connectivity with right amygdala correlated with HRV: HRV+ (across age): Superior Frontal Gyrus (L, R), Middle Frontal Gyrus (L, R). HRV− (across age): Inferior Parietal Lobe (R), Precentral Gyrus (R). HRV+: Young > Old: Globus Pallidus (R), Hypothalamus (R), Superior Temporal Gyrus (R, L), Para-hippocampal Gyrus (R, L), Inferior Frontal Gyrus (L), Insula (L), Cingulate Gyrus (R, L) HRV+: Old > Young: Superior Parietal Lobe (L), Cuneus (L), Precuneus (L). Other regions, which connectivity with left amygdala correlated with HRV: HRV+ (across age): No significant results. HRV− (across age): Cerebellum (R, L), Cuneus (R, L), Lingual Gyrus (L), Precuneus (L), Superior Parietal Lobe (L). HRV+: Young > Old: Para-hippocampal Gyrus (L, R), Superior Temporal Gyrus (L), Inferior Frontal Gyrus (L, R), Middle Temporal Gyrus (L), Inferior Temporal Gyrus (L), Putamen (R), Cingulate Gyrus (R). HRV+: Old > Young: Precentral Gyrus (L, R), Middle Frontal Gyrus (R), Medial Frontal Gyrus (R), Superior Frontal Gyrus (R), Inferior Parietal Lobe (L), Superior Temporal Gyrus (L), Supramarginal Gyrus (L), Inferior Parietal Lobe (L), Middle Temporal Gyrus (L).	+/-	+
		HF	3.0 T fMRI			
McIntosh et. al., 2020 [60]	N = 271 from NKI—RS (62.9% female; aged 18 to 85 years)	(ECG data collected by PPG; 5-min length segments extracted from each IBI series)	(10-min rest period of scan)	HF: Connectivity between the left dlPFC and right MFG associated with greater HF power. Connectivity between the right dlPFC and right SFG and bilateral MFG was associated with greater HF power. Only in women the associations of HF power with the connectivity between left dlPFC and right MFG and right dlPFC and right MFG remains. Analyses performed on a subsample of 232 healthy individuals were consistent with whole-sample findings.	+	+

Definitions for all abbreviations are shown in Table 2.

## 4. Discussion

An increasing number of studies have investigated cortical and subcortical brain regions involved in autonomic control [2,4]. In a meta-analysis performed by Thayer et al. [4], brain regions associated with HRV during different tasks included the left sublenticular extended amygdala/ventral striatum, right sub-genual and pregenual anterior cingulate (regions in the mPFC), right rostral mPFC and left posterior putamen. Another meta-analysis revealed that various brain regions are related to SNS and PNS regulation (assessed as electrodermal activity or related metrics for skin conductivity and HF power, respectively) during different tasks [61]. SNS regulation mostly affects prefrontal, anterior and midcingulate, left posterior and right ventral anterior insular cortices. In contrast, regions of PNS regulation include the posterior CC, bilateral dorsal anterior insular cortices, lateral temporal cortices and PFC activity, including the OFC and mPFC. Some of the regions showed both SNS and PNS regulation, especially the left amygdala and the right inferior parietal lobule [61].

Notably, studies using structural and functional MRI have revealed that thickness, activity and connectivity of some areas of the brain are also related to HRV during baseline rest, before the presentation of different stimuli [32,54]. It has been shown that changes in the activity of the default mode network (DMN), which include structures reflecting higher activation during rest than during a task, correlate with HRV [62]. Similarly, in published studies, although no correlations of HRV with global DMN and SN were observed, some clusters within DMN and SN showed connectivity within the SN or DMN that correlated mostly positively with HF power [59]. Moreover, HRV correlations with activity in brain regions, related to ANS regulation (*i.e.,* the insula, CC, frontal and prefrontal cortices, thalamus, striatum and amygdala) have been reported [38,41,50,51,53,54]. Notably, the CC has been identified as the one of the brain regions whose thickness has the strongest correlations with HRV and the caudal anterior CC was the area of highest associations [2,4,41]. Furthermore, significant correlations between HRV and brain region connectivity (in the amygdala, CC and PFC) were observed. These may suggest that HRV is not directly associated with global resting state activity of intrinsic brain networks, but rather with more localized connectivity [32,59].

Interestingly, vagally mediated HRV was shown to be positively associated with thickness of the right but not left CC region [41]. The lateralization of ANS activity in cardiovascular control has been noted and right-sided brain structures contributing to cardiac vagal control have also been found in studies investigating associations between HRV and brain activity [42,44]. This may suggest that the right-sided neural inputs may be dominant and are especially involved in cardiac chronotropic control via a vagally mediated pathway. However, the evidence for left hemispheric control of cardiac vagal function has also been reported [2,41,50], suggesting that both the right and left hemispheres are involved in PNS control. This is in line with previous observations that nerves on the right side are mainly involved in control of heart rate, while those on the left side contribute to the modulation of atrioventricular conduction [63]. Interestingly, cardio-neuroablation targeting vagal activity through para-cardiac ganglia leads to increased minimum and mean heart rate, decreased SDNN and rMSSD and may lead to treatment of functional atrioventricular block [64].

Age and sex deserve attention because they exert significant effects on central autonomic control and HRV. Studies of differences in heart-brain connections related to ethnicity are rare [56,65]. Older age and aging are believed to be associated with decreased HRV. Research has shown that rMSSD (measured as a 24 h average of 5-min segments) declines approximately 3.6 milliseconds per decade [66]. Moreover, the Cardiovascular Health Study revealed that the frequency-domain HRV parameters decreased most between ages 65–69 and 70–74 years [67]. Interestingly, these associations between age and HRV parameters may be confounded by the increasing presence of sinus arrhythmia of non-respiratory origin which can result in inflated HRV values. There are also essential structural brain changes in aging [68]. The more ventral regions of the brain including the vmPFC and the ACC appear to be relatively maintained with age, while more dorsal, lateral and superior regions show greater decrease in thickness with age [69]. It has also been suggested that age-invariant relationships may exist between HRV and cortical thickness in some brain regions [2]. Results of a study performed by Yoo et al. [2] suggest that age-constant relationships between cortical thickness in more ventral brain regions such as the lateral OFC (regions less related to age-linked decreases in cortical thickness) and HRV may occur. Similarly, a cross-sectional pooled mega-analysis performed by Koenig and Abler [47] revealed that the decline of cortical thickness of the OFC in both hemispheres was most strongly related with the decline in rMSSD. However, a study performed by Wei et al. [42], on a larger group consisting of 185 participants and investigating age effects on the associations of HF power with GMV has shown no significant associations.

When assessing gender differences in the heart–brain connections, the same investigators found that high resting HF power was linked to lower resting CBF in some brain areas (the left para-hippocampal gyrus, left amygdala and right hippocampus), but in females only [56]. Similarly, McIntosh et al. reported that only in women were the associations of HF power with the functional connectivity between left dlPFC and right middle frontal gyrus and right dlPFC and right middle frontal gyrus similar to these observed for the whole group [60]. One of the mechanisms explaining this relationship may be the presence of estrogen. There are several studies investigating the impact of changes in this hormone on brain activity [70,71,72,73]. It was demonstrated that pre-menopausal women have stronger PNS activity, assessed as greater HF power, when compared to women after menopause [74]. Furthermore, it has been shown that PNS activity is stronger in women before menopause when compared to age-matched men. Interestingly, after menopause this association was no longer significant [74,75]. This suggests that estrogen suppresses SNS activation in women before menopause [42,72]. However, a study performed by Wei et al. [42] investigating sex effects on the associations of HF power with GMV showed no significant association (mean age of investigated group was 35.2 ± 14.0 years, 51% were female). Therefore, the effect of menstrual phase on heart–brain connection should be studied.

Additionally, a number of confounding factors should be considered when interpreting HRV measures, e.g., intraindividual variability of HRV, the choice of a physiological signal from which to extract HRV, time of day, body position, body mass index, blood pressure, smoking, alcohol consumption, stress, sleep deprivation, physical activity, different medical conditions, or medication [27,76]. Therefore, it is important to control methodological processes for HRV measurements and try to consider any confounding variables influencing HRV that can be controlled [27,76]. This may be crucial in assuring more accurate and reproducible measures of HRV and avoiding misinterpretation of HRV findings [18]. The studies analyzed in our review control for confounding factors by using specific exclusion criteria consisting of self-reported psychiatric, neurological or medical illness, as well as common exclusion criteria for MRI. However, to optimize HRV measurements, it may be useful and better for patients, instead of using self-report for any psychiatric, neurological, medical illness or blood pressure conditions, to obtain a physical examination by a clinician [27,76].

Dysfunctional brain–heart connections are believed to play an important role in the genesis and progression of disease. Investigation of the heart–brain connection may, therefore, improve our understanding of the mechanisms underlying the relationship between HRV, self-regulatory capacity and both morbidity and mortality [18,41,77]. This is important, because poor capacity for self-regulation may predict worse physical and emotional health and other life problems. Interestingly, relationships between the heart and the brain, and thus possible cardiovascular risk, may be influenced by psychological treatment [78]. However, the correct interpretation of the information that is provided by the various HRV parameters may be difficult. Most studies investigating the role of cortical and subcortical brain regions involved in ANS regulation during rest used HF power or rMSSD as the HRV parameters [2,32,38,41,42,44,50,51,53,58,59]. HF power and rMSSD are assumed in these cases to reflect PNS outflow. Although rMSSD and HF power are highly correlated, rMSSD, as a time-domain measure, may be estimated with less bias and considerably smaller error compared with HF power, which is a frequency-domain parameter [47]. Sympathetic activity cannot be directly derived from HRV analysis. The overlapping activity of both the PNS and SNS activity are influenced by LF power. Generally, higher frequency components of HRV represent PNS influences, especially respiratory sinus arrhythmia, and lower frequencies (< about 0.15 Hz) have a mixture of SNS and PNS influences, but this assumption, without further graphical and non-linear examination of the actual HR patterns, can lead to serious errors in the interpretation of the results [29]. Notably, in a study comparing two bands of LF power, it was revealed that the component of LF power in the 0.1–0.14 Hz band, but not LF power at 0.06–0.1 Hz, correlated with neural BOLD (estimated phase-locking values between precentral gyrus and insula). This may suggest that the component of LF power in the 0.1–0.14 Hz band is related to a PNS influence.

The majority of studies examining the functional links between the brain and heart have explored undirected associations (i.e., rely on correlation-based techniques, consisting of ROI-wise analyses). However, directed casual brain correlates of heart have also been studied using the GCGC [53]. Results suggested the existence of interactions between some brain areas and PNS and SNS regulation. In particular, the amygdala was seen to play an important role in the modulation of HF power. These results appear to be similar to those from correlation-based studies.

An accurate and effective monitoring of biological adaptations and systemic recovery are very important [79]. Results of our study give an insight into neural mechanisms underlying autonomic control and suggest the corroboration of previous findings in the literature that HRV may be useful in clinical practice for assessing, monitoring and interpreting ANS modulation, effectiveness and efficiency. Our results add to the existing findings on the neural concomitants of HRV, because the analyzed studies describe fluctuations of HRV under resting-state conditions, which are crucial in further clinical reasoning [4,8,80]. 

### 4.1. Limitations

Studies described in this review have limitations. One major limitation is that, in most cases, the sample sizes were small and therefore lack generalizability. Thus, the ability of these studies to explore the potential effects of gender and age differences was extremely limited. Optimization of the time window may be difficult, especially in studies assessing HRV and brain regions involved in ANS control during rest [32]. Short windows may not be adequate for the study of all HRV parameters (especially LF power). On the other hand, the sensitivity in detecting temporal changes may be reduced with longer windows. Another potential limitation involves the accuracy of the pulse oximeter which was used in some studies for calculating HRV. With this method, short-term variability measures, such as HF power and rMSSD may be overestimated because the exact timing of the pulse oximeter peak compared to the exact timing of the QRS peak when an ECG is obtained is less clear. However, pulse oximetry is easier to implement and less affected by MRI gradient artifacts than ECG. Most ECG recordings were performed during scans, not before; thus, results may also be affected by MRI-related anxiety. One study assessed state anxiety with the state version of the state-trait anxiety and depression inventory (STADI) and demonstrated that MRI-related anxiety may affect BOLD oscillations and is greater at the beginning of the test. Important also is the fact that the fMRI BOLD signal is modulated both by neuronal and non-neuronal physiological variability (e.g., heart rate, arterial CO2, respiration). However, the resting-state fluctuations of physiological signals such as CO2, respiration and HR are of small magnitude and therefore not expected to significantly affect neuronal activity *per se* [62,81]. Different methods are also used to reduce physiological confounds in the BOLD signal which could result in the removal of components reflecting ANS activity. In two out of the three studies estimating functional connectivity the data were acquired from an approximately 5-min scan which might not be long enough to produce reliable results. Additionally, some of the studies included were conducted by the same group of authors and this could have introduced biases influenced by the theoretical background of those who led the study. 

### 4.2. Future Directions

To overcome these limitations, e.g., in future studies it will be useful to control methodological processes of HRV measurements to ensure more accurate and reproducible measures of HRV and avoiding of the misinterpretation of HRV findings [27,76]. 

Further studies of brain-heart interactions during different state conditions are needed and promise to yield insights into their relationship to health and disease. Moreover, investigation of HRV-biofeedback, which is accompanied by changes in functional brain connectivity during resting state may add significant value in this field [82].

Although this field is in its infancy, it promises to provide insights into brain–heart interactions and their relationship to health and disease and potentially also into pathways by which psychosocial factors can influence them. Umbrella reviews and quantitative analyses, as in other fields of clinical medicine, [83] could be valuable.

## 5. Conclusions

Results of studies performed using magnetic resonance imaging confirm that HRV measures associated with cardiac vagal and sympathetic control can be linked with the volume, activity and connectivity of specific brain regions. Our findings provide support for the interconnection of the brain and the heart by both structural and functional networks and indicate complex multi-level interactions.

## Figures and Tables

**Table 1:** Heart rate variability measures used in studies of brain functioning in neuroimaging studies. Based on: [10,24,29,30,31].

HRV Variables	Definitions	Physiologic Meaning
NN interval (ms)	Time between two consecutive sinus beats.	Reflects both PNS and SNS control of heart rate.
Mean IBI (ms)	Mean inter-beat interval time series (NN interval), refers to the time interval between successive ECG R-wave occurrence times.	Reflects both PNS and SNS control of heart rate.
Time Domain HRV		
SDNN (ms)	Standard deviation of NN intervals.	Reflects overall HRV for period of interest.
rMSSD (ms) (MSSD)	Root mean square of successive variances of NN intervals or the absolute value of the average change in interval between any two normal beats.	When rhythm is normal reflects PNS control of heart rate.
Frequency Domain HRV *#		
LF (ms)^2^	Low frequency power, represents HRV between 0.04 and 0.15 Hz.	Reflects baroreceptor-mediated SNS and PNS impact on heart rate.
HF (ms)^2^	High frequency power, represents HRV between 0.15 and 0.4 Hz.	When rhythm is normal reflects PNS impact on heart rate.
LF/HF ratio	Low frequency power/High frequency power ratio.	Often interpreted as indicative of SNS to PNS balance, interpretation of this index is controversial [29,30].
LF (nu)	Normalized low frequency power, represents the proportion of total HRV that occurs in the LF band.	Often interpreted as indicative of SNS activity, but interpretation of this index is controversial [29,31].
HF (nu)	Normalized high frequency power, represents the proportion of total HRV that occurs in the HF band.	Reflects PNS activity.
Non-Linear Measurements		
ipApEn	Inhomogeneous point-process approximate.	Measures the regularity and complexity of a time series.
ipSampEn	Sample entropy.	Measures the regularity and complexity of a time series.
IDLE	Instantaneous dominant Lyapunov exponents.	Measures a non-linear system’s sensitive dependence on starting conditions.
SD1	Poincaré plot short axis of an ellipse fitted to plots.	Reflects short-term HRV, identical to rMSSD parameter.

* Depending on study, these parameters were expressed in original units or as the natural logarithm (Ln). # In most studies, the recordings lasted <5 min, but not all researchers reported the recording duration. The recording duration was 10 min in two studies [32,33]. Abbreviations: ECG—electrocardiogram; HRV—heart rate variability; NN—normal-to-normal; PNS—parasympathetic nervous system; SNS—sympathetic nervous system.

## Data Availability

Not applicable.
